# Frequency of impulse control behaviours associated with dopaminergic therapy in restless legs syndrome

**DOI:** 10.1186/1471-2377-11-117

**Published:** 2011-09-28

**Authors:** Valerie Voon, Andrea Schoerling, Sascha Wenzel, Vindhya Ekanayake, Julia Reiff, Claudia Trenkwalder, Friederike Sixel-Döring

**Affiliations:** 1 Behavioural and Clinical Neurosciences Institute, University of Cambridge, Cambridge, UK; 2 Paracelsus-Elena-Hospital, Center of Parkinsonism and Movement Disorders, Kassel, Germany; 3 Department of Psychological Sciences, Purdue University, West Lafayette, IN, USA; 4 Klinik für Psychiatrie und Psychotherapie, Dr. Horst Schmidt Klinik, Wiesbaden, Germany

**Keywords:** Restless legs syndrome, impulse control disorders, dopamine agonist, gambling, levodopa

## Abstract

**Background:**

Low doses of dopamine agonists (DA) and levodopa are effective in the treatment of restless legs syndrome (RLS). A range of impulse control and compulsive behaviours (ICBs) have been reported following the use of DAs and levodopa in patients with Parkinson's disease. With this study we sought to assess the cross-sectional prevalence of impulse control behaviours (ICBs) in restless legs syndrome (RLS) and to determine factors associated with ICBs in a population cohort in Germany.

**Methods:**

Several questionnaires based on validated and previously used instruments for assessment of ICBs were mailed out to patients being treated for RLS. Final diagnoses of ICBs were based on stringent diagnostic criteria after psychiatric interviews were performed.

**Results:**

10/140 RLS patients of a clinical cohort (7.1%) were finally diagnosed with ICBs, 8 of 10 on dopamine agonist (DA) therapy, 2 of 10 on levodopa. 8 of the 10 affected patients showed more than one type of abnormal behaviour. Among those who responded to the questionnaires 6/140 [4.3%] revealed binge eating, 5/140 [3.6%] compulsive shopping, 3/140 [2.1%] pathological gambling, 3/140 [2.1%] punding, and 2/140 [1.4%] hypersexuality in psychiatric assessments. Among those who did not respond to questionnaires, 32 were randomly selected and interviewed: only 1 patient showed positive criteria of ICBs with compulsive shopping and binge eating. ICBs were associated with higher DA dose (p = 0.001), younger RLS onset (p = 0.04), history of experimental drug use (p = 0.002), female gender (p = 0.04) and a family history of gambling disorders (p = 0.02), which accounted for 52% of the risk variance.

**Conclusion:**

RLS patients treated with dopaminergic agents and dopamine agonists in particular, should be forewarned of potential side effects. A careful history of risk factors should be taken.

## Background

An increasing awareness of restless legs syndrome (RLS) [1] has led to a higher number of patients being treated with dopaminergic agents. Although a dopaminergic deficit has not been demonstrated in RLS [2], it is believed that an imbalance of the diencephalo-spinal dopaminergic system exists [3] and that it interacts with the iron metabolism [4]. Low doses of dopamine agonists (DA) are effective for improving sleep disturbances, daytime symptoms and quality of life of RLS sufferers [5]. The DAs ropinirole, pramipexole and rotigotine transdermal patch are licensed for treating RLS in most European countries and--except for rotigotine--in the US. In Germany, Austria and Switzerland levodopa/benserazide has also received regulatory approval for RLS therapy.

A range of impulse control and compulsive behaviours (ICBs) have been reported following the use of DAs and levodopa in patients with Parkinson's disease. DAs are particularly associated with pathological gambling, compulsive shopping, hypersexuality and binge eating [6-10]. Systematic studies on pathological gambling have been conducted in Italy [7] and the United Kingdom [9] but none have been reported in Germany. Hypersexuality has been reported with levodopa monotherapy, although it is more likely to occur on DAs. Binge eating has also been associated with DAs [10,11]. Punding has been variably associated with levodopa and DAs [12-14]. Compulsive medication use has been associated with levodopa [15,16]. Such behaviours are variably associated with earlier age of disease onset, a personal or family history of gambling or alcohol use disorders, smoking, greater experimental drug use, and higher novelty seeking and impulsivity scores [10,14,15,17,18]. The objectives of this study were to assess the cross-sectional prevalence of ICBs in RLS using stringent diagnostic criteria and to determine factors associated with ICBs in a population cohort in Germany.

## Methods

The questionnaires used in this study were based on different screening tools that had either been validated in the general population or had been used in previous studies of ICBs in Parkinson's disease. These screening tools were:

1. The South Oaks Gambling Screen was used to assess gambling [19].

2. A previously published questionnaire for Parkinson's disease was used for hypersexuality [6].

3. The Minnesota Impulse Disorders Interview was used for compulsive shopping [8].

4. Questions adapted from Evans et al.'s questionnaire were used for punding [14].

Associated factors that were also assessed included experimental drug use, cigarette smoking, alcohol use and family history of alcohol/substance use and gambling disorders, and novelty seeking behaviours. The questionnaires were translated into German following recommended rules for translating scales with back translation from two different translators, and mailed out to all RLS patients who had consulted at the RLS outpatient clinic of the Paracelsus-Elena Hospital in Kassel, Germany, within the previous two years.

All patients were interviewed by an RLS expert (CT) and their diagnosis confirmed according to the essential RLS diagnostic criteria, a full history was performed and a neurological exam made. Patients with low ferritin levels were included in the sample; ureamic RLS and other secondary causes of RLS were excluded.

A cut-off for the screening questionnaires was defined according to previous studies [20]. Patients who scored above cut-off on the screening questions were contacted and extensively interviewed in person in the clinic or by phone by either a psychologist (AS) or a psychiatrist (SW), using established or previously used stringent diagnostic criteria [6,14,16,21,22], reviewed in [20]. The study was approved by the Ethics Board of the Landesaerztekammer Hessen in Wiesbaden, Germany.

### Statistical analysis

We used the Fisher's Exact Test and unpaired t-tests for categorical and continuous variables respectively, and Spearman's rank test for non-parametric variables (cigarette, alcohol use) was used with SPSS Version 16.0. Since the factors tested were based on known associations with ICBs in Parkinson's disease, p < 0.05 was considered significant.

## Results

**Figure 1:**
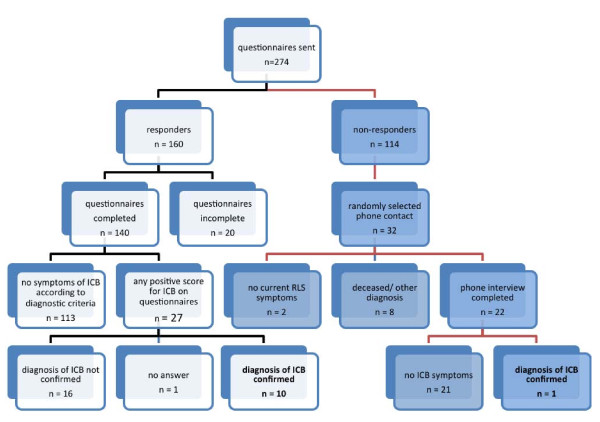
**Study tree of 274 RLS patients**.

The study tree is shown in Figure 1. Fifty-seven percent (160/274) of subjects responded to the mail-out questionnaires. Face-to-face clinical interviews or extensive phone interviews were conducted with 27 subjects who showed any positive score on the mail-out questionnaires. One-third (32/114) of the non-responders were randomly selected for contact by telephone (CT). Subjects commented that they did not respond to the questionnaires as they had either never experienced any of these behaviours, felt irritated by the questions, or did not require treatment for RLS symptoms at that time.

ICBs were reported in 10/140 (7.1%) respondents after an extensive interview (Table 1) and 1/32 non-respondents (compulsive shopping and binge eating) contacted by phone. Of the responders, compulsive eating was identified in 6/140 (4.3%), compulsive shopping in 5/140 (3.6%), pathological gambling in 3/140 (2.1%), punding in 3/140 (2.1%), hypersexuality in 2/140 (1.4%) and compulsive medication use in none. Subjects with computer gambling spent money gambling on the Internet and fulfilled criteria for pathological gambling. Two subjects on levodopa monotherapy developed compulsive shopping in addition to comorbid punding in one subject. All patients had ICB onset after medication initiation, none had previously been identified as having an ICB.

**Table 1:** Impulse control and compulsive behaviours diagnoses and medication data in patients with restless legs syndrome

Patient	ICB diagnosis	DA type/dose	L-dopa dose
1	Compulsive shopping, binge eating	Ropinirole/2mg	100 mg

2	Computer gambling, hypersexuality	Pramipexole/0.72 mg	--

3	Binge eating, punding	Pramipexole/1.4 mg	--

4	Gambling, binge eating	Ropinirole/4.5 mg	--

5	Compulsive shopping, binge eating	Ropinirole/2 mg	--

6	Computer gambling, hypersexuality, binge eating	Pramipexole/0.95 mg	--

7	Compulsive shopping, binge eating	Lisuride/2.5 mg	--

8	Punding	Cabergoline/3 mg	

9	Compulsive shopping	--	100 mg

10	Compulsive shopping, punding	--	500 mg

All ten subjects identified with an ICB were female. Female-specific prevalence rates were as follows: compulsive eating in 6/98 (6.1%), compulsive shopping in 5/98 (5.1%), pathological gambling in 3/98 (3.1%), punding in 3/98 (3.1%) and hypersexuality in 2/98 (2.0%). ICBs were reported in 10% of women who responded to the questionnaire. Associated factors are reported in Table 2. All subjects with ICBs received dopaminergic therapy. In the RLS group without ICBs 122/130 subjects (93.8%) received dopaminergic therapy, 8/130 subjects (6.2%) received only non-dopaminergic medication. ICBs were associated with higher DA dose, younger age of RLS onset, history of experimental drug use, female gender and a family history of gambling disorders (Table 2). Using a logistic regression analysis, these factors accounted for 52% of the variance and predicted ICBs at 42.9%. P-values at model entry were as follows: DA dose (p = 0.002), experimental drug use (p =< 0.0001), age of RLS onset (p = 0.05), gender (p = 0.12). The addition of female gender to the model increased the R^2 ^value from 45 to 52 and was thus included in the model.

**Table 2:** Patient characteristics and factors associated with impulse control and compulsive behaviours in patients with restless legs syndrome

		RLS + ICBs	RLSnonICBs	p-value
**Total number**		n = 10	n = 130	

**Female gender**		n = 10 (100%)	n = 88 (67.6%)	**0.04**

**Mean age of RLS onset in years (SD)**		46.6 (10.1)	57 (15.9)	**0.04**

**Medication**	Dopaminergic	n = 10 (100%)	n = 122 (93.8%)	0.4

	Other	n = 0 (0%)	n = 8 (6.2%)	

**Da type**	Ropinirole	n = 3 (37.5%)	n = 29 (29.8%)	0.6

	Pramipexole	n = 3 (37.5%)	n = 43 (44.3%)	0.5

	Other	n = 2 (25%)	n = 25 (25.7%)	0.3

Mean DA dose as LEDD mg/d *(SD)		63.7 (52.7)	26.7 (26.4)	**0.001**

**FHx alcohol use disorder**		n = 3 (30%)	n = 12 (9.2%)	0.09

**FHx gambling disorder**		n = 2 (20%)	n = 2 (2%)	**0.02**

**Hx experimental drug use**		n = 5 (50%)	n = 10 (7.9%)	**0.002**

**Cigarettes smoked daily**	0	n = 5 (50%)	n = 81 (62.3%)	0.19

	1-10	n = 0 (0%)	n = 10 (13%)	

	11-20	n = 3 (30%)	n = 13 (10%)	

	21-29	n = 0 (0%)	n = 6 (6%)	

	> 30	n = 2 (20%)	n = 10 (13%)	

**Alcohol daily glasses**	0	n = 8 (80%)	n = 70/118 (59.3%)	0.18

	1-7	n = 2 (20%)	n = 41/118 (34.8%)	

	8-14	n = 0 (0%)	n = 5/118 (4.2%)	

	15-21	n = 0 (0%)	n = 2/118 (1.7%)	

	> 22	n = 0 (0%)	n = 0 (0%)	

**Mean novelty seeking score (SD)**		20.8 (5.3)	18 (5.5)	0.13

*Calculation of Levodopa Daily Dose Equivalent (23); Abbreviations: RLS = Restless Legs Syndrome; ICBs = Impulse control behaviors; SD = standard deviation; DA = dopamine agonist; LEDD = levodopa dose equivalent; FHx = Family history; Hx = history; cut-off for significance: p < 0.05

## Discussion

ICBs occurred in 7.1% (10/140) of RLS patients treated with either a DA (8/10) or levodopa monotherapy (2/10). Eight of ten (80%) subjects had more than one comorbid ICB. ICBs in RLS patients were associated with higher levodopa-equivalent DA dose [23], young age of RLS onset, history of experimental drug use, female gender and a family history of gambling disorders. These factors also accounted for 52% of the variance and predicted ICBs at 42.9%.

Study strengths include the use of validated or previously utilized screening questionnaires and established instruments to assess associated factors, as well as the use of stringent diagnostic criteria and extensive interviews with a psychologist or a psychiatrist. Based on the identification of ICBs in only 1/32 randomly selected non-responders (3.1%) we caution that the occurrence of ICBs in RLS patients found in this study may be over-estimated, as the experience of behavioural changes may have motivated the subjects to fill out the questionnaires and thus bias the results of the study.

One limitation of the study is the lack of a control group. However, all RLS patients with ICBs were on dopaminergic therapy and none of the RLS patients with non-dopaminergic medication was identified with an ICB. In accordance with previous findings [24-27] this is supportive of an association between ICBs and dopaminergic medication in RLS patients. We can also confirm the previous association between the occurrence of ICBs in RLS and treatment with higher dosages of dopaminergic agents compared to RLS patients who did not develop ICBs [24,26]. As depression may present an individual susceptibility factor for abnormal behaviours in PD patients [28] and neuropsychological testing revealed preserved executive functions in a previous study on PD patients with pathological gambling [29], the lack of further psychiatric and neuropsychological evaluation in our cohort further limits the study presented here. Comparative assessment of psychiatric comorbidities and cognitive profiles should be addressed in further studies.

Compulsive eating was identified in 6/140 (4.3%), compulsive shopping in 5/140 (3.6%), pathological gambling in 3/140 (2.1%), punding in 3/140 (2.1%), hypersexuality in 2/140 (1.4%) and compulsive medication use in none of the subjects. These frequencies are relatively similar to RLS studies conducted in North America [24-27] although the phenotypes of ICB are different compared to US patient cohorts. One study compared 100 RLS treated patients, 275 controls with obstructive sleep apnoea and 52 RLS untreated patients. RLS patients on dopaminergic medications had greater pathologic gambling (5%), compulsive shopping (9%) and punding (7%) than the sleep apnoea control group along with greater compulsive shopping relative to the RLS untreated group [24,26]. Our study uniquely focuses on ICBs in RLS patients in a European rather than a North American cohort and further investigates associated factors. The frequency of ICBs is greater in Parkinson's disease compared to RLS and has been suggested to be related to dose effects [24]. In a large multicentre North American study focusing on Parkinson's disease, pathological gambling is reported in 2.9% (with problem gambling in an additional 2.3%), compulsive shopping in 6.0%, hypersexuality in 3.5%, and binge eating in 3.5% [10]. Punding has been reported in 1.5 to 14% [12,14] and compulsive medication use in 3 - 4% [16,30].

In the North American general population, binge eating disorder is identified in 3.5% of women and 2% of men [31] and compulsive shopping in 6% [32]. The lifetime prevalence of a binge eating disorder in European countries, including Germany, has been reported to be 1.12% [33]. The lifetime prevalence of pathological gambling in North America is 1.5% [34] whereas the 2006 German Epidemiological Survey of Substance Abuse (n = 7817, aged 18-64 years old), that was controlled for mania, found a much lower lifetime prevalence of DSM IV-diagnosis of pathological gambling at 0.09% [35]. Pathological gambling in Germany may be lower due to a negative cultural attitude towards gambling as well as a less widespread access to casinos. Intriguingly, the rate of pathological gambling in RLS is similar to the rate reported in PD in North America, but access to gambling in our study in Germany was through the Internet. The prevalence rates of binge eating and compulsive shopping in RLS patients may reflect a different pattern of presentation in females. We did not find RLS patients with compulsive medication use, which may be due to the lower frequency and dosage of levodopa use in Germany.

Associated factors such as a history of experimental drug use and a family history of gambling disorders are consistent to that observed in the literature on ICBs in Parkinson's disease [10,14,15,17]. Factors such as cigarette and alcohol use and novelty seeking may reach significance with a larger sample size. This suggests that a similar biological predisposition may underlie these behaviours rather than Parkinson's disease itself being an absolutely necessary factor. The young age of RLS onset suggests that genetically mediated forms of RLS may have a different susceptibility to dopaminergic medications. In Parkinson's disease, male gender is considered a risk factor for specific ICBs such as hypersexuality [36]. In our study only women were affected by ICBs. Further studies in large cohorts are necessary to clarify whether this is due to the greater overall prevalence of RLS in women - as reflected in our study cohort.

None of the RLS patients identified with ICBs in this study attributed their ICB symptoms to dopaminergic treatment. Although all affected study subjects were advised to have their treatment regimen changed, none felt sufficiently impaired by their behaviours to stop dopaminergic medications completely, but agreed to a reduced dosage or combination therapy with non-dopaminergic agents.

## Conclusions

Higher DA dose, young age of RLS onset, history of experimental drug use, female gender and a family history of gambling disorders may be predisposing factors for developing an ICB in RLS with dopaminergic treatment. RLS patients should be forewarned of potential behavioural side-effects when treated with dopaminergic agents and dopamine agonists in particular.

## Competing interests

The authors declare that they have no competing interests.

## Authors' contributions

VV participated in the conception, design, organization and execution of the study, designed and critically reviewed the statistical analysis, wrote the first draft of the manuscript and critically reviewed the revised version. AS participated in the organization of the study and conduction of the patient interviews. SW participated in the design and organization of the study, conduction of the patient interviews and was responsible for confirmation of ICB diagnoses. VE performed the statistical analysis. JD participated in the design of the study and critically reviewed the manuscript. CT participated in the conception, design, organization and execution of the study, critically reviewed statistical analysis, participated in the writing of the first draft and critically reviewed the manuscript as well as its revised version. FSD conceived the study, participated in its execution, critically reviewed the first draft and wrote the revised version. All authors read and approved the final manuscript.

## Pre-publication history

The pre-publication history for this paper can be accessed here:

http://www.biomedcentral.com/1471-2377/11/117/prepub
